# A Rare Presentation of Shared Phenomenon in Dissociative Disorders in Extreme of Ages: A Report of Two Cases

**DOI:** 10.7759/cureus.32911

**Published:** 2022-12-25

**Authors:** Anurag Sengar, Rajiv Mehta, Oluwasayo J Owolabi, Tulika Garg, Uchenna E Ezenagu, Esther O Apata, Munira Abdefatah Ali, Zainab Omar, Hassan A Chaudhry, Aadil Khan

**Affiliations:** 1 Psychiatry, Sir Gangaram Hospital, New Delhi, IND; 2 Psychiatry, Lugansk State Medical University, Lugansk, UKR; 3 Medicine, Government Medical College and Hospital, Chandigarh, IND; 4 Medicine, Sumy State University, Sumy, UKR; 5 Psychiatry, Lugansk State Medical University, Luhansk, UKR; 6 College of Medicine, University of Illinois Chicago, Chicago, USA; 7 Pediatrics, Dubai Medical College For Girls, Dubai, ARE; 8 Biological Sciences, Temple University, Philadelphia, USA; 9 Medical School, Medical University of Lublin, Lublin, POL; 10 Interdisciplinary Medicine, Independent Research Scholar, Philadelphia, USA; 11 Internal Medicine, Lala Lajpat Rai (LLR) Hospital, Kanpur, IND

**Keywords:** depersonalization disorder, dissociative identity disorder, generalized anxiety disorder, pseudoseizures, conversion disorders

## Abstract

Conversion disorders (CD) are changes in sensorimotor activity experienced by an individual due to an external event. Patients may experience "pseudoseizures" accompanied by the presence or absence of loss of consciousness. Disorders of movement and sensation is the term used to classify the various kinds of CDs in the International Classification of Diseases, Tenth Revision (ICD-10) diagnostic manual, and they are the rarest among all dissociative disorders. We will discuss two instances that are particularly rare. The first includes an older couple, starting with the wife, who had nervousness, heightened worry, intrusive thoughts, heavy perspiration, palpitations, headaches, and problems sleeping. She was prescribed 10 mg once-daily escitalopram. She stopped taking her medication and had facial and hand problems. The patient's 65-year-old husband started having strange hand and face movements and lost consciousness. The pair was hospitalized willingly and had radiographic (MRI and non-contrast computerized tomography {NCCT} head), nerve conduction, and neurological tests to rule out a movement issue. No inquiry or inspections uncovered anything unusual. The second case involves a mother and her 13-year-old son, who was taken to a psychiatric unit after urinating on a religious shrine. His mother had the same issue and couldn't urinate for days. Both patients were given 25 mg of paroxetine and benzodiazepines for anxiety and sleeplessness. After a week of medicine and psychotherapy after identifying stressors, both cases improved.

## Introduction

True conversion disorder is uncommon, affecting between two and 22 people per 100,000 people annually [1]. It may be more prevalent among military personnel, rural residents, and people with a lower socioeconomic status. It is unknown how frequently it affects men and women, although the disease is more common among adolescents than it is among children [2,3]. One or more conversion disorder symptoms impair conscious motor or sensory function, indicating a neurological or other physiological disorder. Nonetheless, these symptoms are inconsistent with known neurological or musculoskeletal disorders [4]. Individuals with conversion disorders (CD) do not intentionally develop or imitate their symptoms. Instead, the symptoms are caused by an unconscious psychological conflict or desire. Symptoms are frequently exacerbated by social interference from family and friends or by emotional stress avoidance. One or more limbs becoming paralyzed, ataxia, tremors, tics, and dystonia are only a few of the conversion disease's debilitating symptoms [5]. Functional gait disorder, hysterical paralysis, psychosomatic disorder, conversion response, and persistent neurosis are additional labels for this syndrome [6]. There may be primary and secondary gains that function as sustaining factors. CDs continue to be poorly understood and difficult to diagnose despite their well-documented history. It is frequently misdiagnosed as other neurological and psychiatric disorders, creating diagnostic difficulties for physicians. Identification of these patients immediately is crucial [7].

## Case presentation

Case 1: a shared dissociative disorder between elderly married couple

We present a case of a 61-year-old female who came to the outpatient department (OPD) of the psychiatry unit, at a tertiary care hospital, with a chief complaint of apprehension, increased worries and thoughts, excessive sweating, and palpitations. In addition, she also had somatic complaints like frequent headaches, aches, pain all over the body, and decreased sleep for the past seven months. On the integration of the patient's symptoms, we diagnosed her with generalized anxiety disorder (GAD) according to the International Classification of Diseases, Tenth Revision (ICD-10) classification. As a result, she was started on escitalopram 10 mg once a day, along with benzodiazepines. On the follow-up appointment, the patient's condition improved significantly after one month of straight medication.

Two months later, she discontinued her medication and had a significant psychosocial stressor during this duration. She again presented to psychiatric OPD with a chief complaint of abnormal body movement in her hands and face. She also had a few episodes of unconsciousness, not associated with abnormal body movement, up-rolling eyeballs, or tongue bites. No urinary or fecal incontinence was documented. Moreover, after a few days, her husband, a 65-year-old elderly male, started complaining of abnormal body movement in his hands and face and episodes of loss of consciousness. Voluntary admission of both patients was made. Subsequently, we performed various routine and radiological investigations (MRI, non-contrast computerized tomography {NCCT} head), nerve conduction study (NCS), and a complete neurological examination to rule out any underlying neurological movement disorder. However, all investigations and assessments were unremarkable. Upon integrating their signs and symptoms, we concluded the diagnosis of dissociative disorders of movement and sensation according to ICD-10. For both patients, we started the appropriate pharmacotherapy, which consisted of paroxetine controlled-release tablets 25 mg once a day, and benzodiazepine for anxiety and sleep disturbance management. After one week of treatment, we identified multiple stressors and prescribed short-term psychotherapy. Both patients responded well to treatment and showed similar alleviation of symptoms.

Case 2: a shared dissociative disorder between mother and adolescent son

We present the case of a 13-year-old child who was referred to a psychiatric unit from a tertiary care hospital pediatric unit. He presented to the pediatric team with lower limb weakness associated with pain for 10 days. In addition, he was unable to walk on his foot. Instead, he was sliding in a peculiar gait (squatting position) for two days. Owing to this, he was treated symptomatically by the pediatric unit; however, he did not respond to the treatment given hence he was referred to a psychiatric unit. He underwent routine radiological and nerve conduction studies in the pediatric unit to exclude any organic neurological causes. Subsequently, all the investigations came up with normal results.

His mental status examination and psychological assessment were done in the psychiatric unit. After further history exploration, it was found that his 35-year-old mother had the same complaint of lower limb weakness associated with pain and sliding in a squatting position. So, his mother was also called for an assessment in the psychiatry OPD to understand the case better. On further exploration of history, we learned that the boy urinated at a shrine a few days back and complained of pain in his lower limbs. The mother correlated the event that since the boy had urinated at the shrine, he developed pain. She went to the shrine along with her child and performed some rituals so that the departed soul might forgive her child. The boy continued to experience pain, following which his mother started to experience lower limb weakness associated with severe diffused lower limb pain and abnormal gait. In addition, she did not void urine for one day. She would think that all her symptoms are due to the abovementioned event. Moreover, on physical examination of the mother, bulging in the lower abdomen was identified because she could withhold urine for long time.

After combining clinical signs and symptoms, both mother and son were diagnosed with dissociative disorders of movement and sensation according to ICD-10. Additionally, the mother had anxiety symptoms like palpitations, sweating, and dizziness with decreased sleep. Subsequently, both mother and child were voluntarily admitted to the unit, and the mother was started on paroxetine 25 mg once a day, accompanied by benzodiazepines for anxiety symptoms. The child was not begun on any pharmacotherapy, instead relaxation techniques and psychotherapy were initiated. After 10 days of therapeutic intervention, patients were given family-focused cognitive behavior therapy. Both patients responded well to the treatment and were discharged and followed up.

## Discussion

Previously, CD and dissociative disorders, such as "functional" or "psychogenic" amnesia, dissociative identity disorder (DID), fugue, and depersonalization disorder, were classified as forms of hysteria. In industrialized nations, 2.4% of the population suffers from dissociative disorders characterized by a disruption of normally integrated functions. It is believed that traumatic events, intolerable stressors, or dysfunctional relationships cause psychogenic dissociative disorders [8]. As a result, assumptions can be made regarding how the individual copes with their insoluble distress.

In addition to the essential diagnostic indicators, patients with DID typically exhibit additional symptoms [9]. Typically, dissociative states resolve within weeks or months, especially if triggered by a traumatic event. If they are associated with intolerable problems or interpersonal issues, chronic conditions such as paralysis and anesthesia may develop (sometimes more slowly). People with dissociative disorders frequently deny the existence of problems or difficulties that may be apparent to others. Consequently, the patient may present as having a physical disorder despite the fact that no physical disorder can be identified as the cause of the symptoms.

Furthermore, an evaluation of the patient's mental state and social situation typically indicates that the disabled state aids the patient in avoiding unpleasant conflicts and expressing dependence or resentment. In addition to a central, constant, and involuntary loss of movement or sensation, a small amount of attention-seeking behavior may also be present. Some patients exhibit symptoms that are strongly associated with psychological stress, whereas others do not. Personal relationship and personality abnormalities are typically present, and close relatives and friends may have experienced similar physical illnesses. Mild and transient forms of these disorders are prevalent during adolescence, particularly among girls, whereas chronic forms are prevalent among young adults. Some elderly individuals who consistently respond to stress, in the same way, may still have these disorders [10].

In all cultural contexts, dissociation and coping mechanisms are prevalent [11]. Studies from Turkey, Scandinavia, Canada, Australia, the United States, Ireland, and the United Kingdom suggest that dissociative and cognitive dysfunctions may share similar brain dysfunctional processes [12]. The orbitofrontal cortex, thalamus, anterior cingulate cortex, insula, and ventrolateral prefrontal cortex are all associated with CD [13]. The right cerebellum, right orbitofrontal cortex, left putamen, and left thalamus were all implicated in a functional MRI (fMRI) investigation of hypnotically induced paralysis, and hypnotizable people show substantial functional connections between their dorsolateral and frontal prefrontal cortex [14,15].

## Conclusions

The cases described here are uncommon presentations of dissociative disorders of movement and sensation in which the patients exhibited identical symptoms. The cases are unique in that they are observed at extreme ages. In the first case, a 55-year-old woman shared her symptoms with her 61-year-old husband; in the second case, a 35-year-old mother and her 13-year-old child had identical symptoms. In our cases, the symptoms of dissociative disorders are frequently very similar in close relationships. The most intriguing aspect of both cases is that the patient's symptoms began diminishing simultaneously and in a similar manner. Both patients in the first case received pharmacotherapy in addition to psychotherapy, whereas the child in the second case was managed solely through psychotherapy.

CD, also known as functional neurological symptom disorder, is a complex illness characterized by symptoms that manifest in both physical and mental health conditions. It can also be exacerbated by the social environment of the patient. Early diagnosis and treatment can prevent the need for unnecessary tests and treatments. Before making this diagnosis, a comprehensive medical evaluation is required to ensure that no underlying medical condition is overlooked. Early detection of potential CD would facilitate referral to the appropriate specialist, as many individuals with conversion symptoms initially seek treatment in primary care settings. In addition to patient interviews and medical evaluations, psychological and neuropsychological assessments can be valuable tools (for example, physical examinations, neuroimaging, and laboratory tests). Early CD detection enables early CD treatment, delaying the onset of symptoms as much as possible.
